# Mix and Match: An Investigation into Whether Episodic Future Thinking Cues Need to Match Discounting Delays in Order to Be Effective

**DOI:** 10.3390/bs9010001

**Published:** 2018-12-21

**Authors:** Sara O’Donnell, Kelseanna Hollis-Hansen, Leonard H. Epstein

**Affiliations:** Department of Pediatrics, Jacobs School of Medicine and Biomedical Sciences, University at Buffalo, New York, NY 14214-3000, USA; kasmith@buffalo.edu (K.H.-H.); lhenet@buffalo.edu (L.H.E.)

**Keywords:** episodic future thinking, delay discounting, temporal

## Abstract

Episodic future thinking (EFT), or prospectively imagining yourself in the future, has been developed into an intervention tool to reduce delay discounting (DD), or the preference for smaller immediate over larger future rewards, and to make healthier choices that promote long-term health rather than short-term enjoyment. Most EFT interventions use EFT cues whose future events match the time delays of the DD task, which may limit the utility of EFT. The current study (*N* = 160, *M*_age_ = 35.25, 47.5% female) used a 2 × 2 factorial design with type of episodic thinking (matched, unmatched) and temporal perspective (EFT, episodic recent thinking (ERT)) as between-subject factors to investigate whether there were differences in DD for groups that had EFT cues matched to the time delays of the DD task in comparison to cues with unmatched temporal delays. The results showed EFT reduced DD compared to ERT controls, and no differences emerged between matched and unmatched EFT groups. Our findings suggest that either the process of generating EFT cues or the use of any positive and vivid future event, regardless of whether it is matched to the DD task, can reduce DD.

## 1. Introduction

Many everyday decisions involve choices between rewards available immediately, or larger rewards available to us in the future. It can be difficult to make choices that result in decreased pleasure in the moment in order to wait for a larger future reward. For example, going out to dinner after a long day at work may sound tempting, but it is a choice that comes at the expense of putting that money into a savings account. This bias toward smaller immediate rewards over larger future rewards is delay discounting (DD), and it increases the longer the time required to wait for a future reward [1]. The habitual preference for smaller immediate rewards characterizes many maladaptive behaviors, such as overeating [2,3], alcohol and drug use [4,5], and problematic gambling [6]. 

Because immediate bias is implicated in so many problematic behaviors, researchers have begun to investigate interventions to reduce DD. One such intervention is episodic future thinking (EFT). EFT is a type of prospective memory that involves vividly imagining oneself engaging in behavior at a future event and is a skill that is often used for future planning and decision-making [7]. People are asked to imagine the setting they are in, the people that are present, what behaviors they are doing, and what is going on around them [8]. Researchers have harnessed this natural ability of our memory to develop an intervention that has reduced DD in children [9] and adults [8,10,11]. Research suggests that EFT decreases the bias toward immediate gratification by increasing a personal connection toward the future and improving the valuation [12] or cognitive salience of the delayed reward [13]. 

To engage in EFT, participants are asked to create “EFT cues” about future events that occur at the same time as the time delays of a hypothetical monetary discounting task. For example, if a DD task encompasses decisions between smaller amounts of money now and larger amounts of money at seven future time points (e.g., 1 day, 2 days, 1 week, 2 weeks, 1 month, 6 months, and 2 years from today), then individuals would be asked to generate positive and vivid events for each of those seven time periods (e.g., “In 1 day I am on a walk at Delaware Park with my best friend Tierney”). These “EFT cues” are matched to temporal windows assessed in the DD task.

Investigating the optimal EFT protocol is relevant to the application of EFT interventions designed to improve behaviors associated with high DD. EFT cues have reduced alcohol reinforcement [14], energy intake during an ad-lib eating scenario [9], and food reinforcement [15]. Researchers have found that tailoring EFT cues to match the environmental context of the decision can amplify its effect on DD [11]. In this study, EFT cues that included the financial components of future events improved monetary DD greater than nonfinancial EFT cues. This suggests that EFT interventions may work best when the content of the cues matches the content of the reward (e.g., EFT cues to reduce overeating including a health component). 

An important methodological consideration is whether the time periods of EFT cues need to match the time periods of the delay-discounting task. It is logical that the initial studies on EFT did match the time periods of the cues with the time delays, but it is unknown whether the effect of EFT depends on this matching, or whether the effect of EFT is based on engaging in prospective thinking. Preliminary evidence has shown that engaging in EFT using only a single EFT cue did not reduce DD, while engaging in EFT with multiple future event cues did reduce DD [16]. This study had participants either create one EFT cue, corresponding to the longest time delay of the DD task, or three EFT cues, which were approximately matched to the time delays of the DD task. It is unknown whether the lack of an effect for a single EFT cue was due to the lack of correspondence between the cue and the time delays of the task, or because a single EFT cue is insufficient to shift discounting rates. It is possible that the process of creating multiple EFT cues primes the decision-maker to make choices that benefit the future self and multiple cues are needed to adequately prime the individual for an optimal effect of EFT.

The current study sought to extend previous research and investigate whether EFT cues matched to the time delays of the delay-discounting task were more powerful at shifting delay-discounting rates than randomly paired EFT cues. Matched and unmatched episodic recent thinking (ERT) cues were used as controls. 

## 2. Methods

### 2.1. Participants

Participants (*N* = 160) were recruited through Amazon Mechanical Turk (MTurk), a crowdsourcing Internet marketplace where small tasks are posted as human intelligence tasks (HITs) for human workers to complete. Only MTurk workers who had a 95% acceptance rate on previous HITs and who currently resided in the United States could access the survey. Thirty-one percent of the participants were minority, 48% were female, and the average age was 35.25 (SD = 9.42). All participants had graduated high school, with 79% having completed at least one year of college education. Participants were excluded from completing the survey if they endorsed tobacco or illicit substance use (e.g., opiates, stimulants) with the exception of marijuana, or if they were not at least 18 years old. There are two reasons why we decided to include marijuana users while not including other drugs of abuse. First, while drug use is typically associated with greater delay discounting, that is not the case with marijuana. For example, tobacco [17,18], cocaine [19], and opioid [20] users have higher discounting than non-users. However, delay discounting does not differ between users and non-users of marijuana [21], suggesting that including marijuana users would not influence our results. Second, our study relied on Amazon’s Mechanical Turk to collect online data from individuals across the United States. Currently, 22 states have decriminalized or legalized marijuana [22], and we could have lost a significant number of potential participants if we excluded marijuana use, in addition to limiting the generalizability of our results. For these reasons, we did not exclude marijuana users from participation.

Individuals were told they were participating in a study that sought to examine factors that influence decision-making. Participants were paid $1.00 for completing the survey and awarded a $5.00 bonus if they followed instructions and carefully completed 100% of the survey questions. All subjects gave their informed consent for inclusion before they participated in the study. The study was conducted in accordance with the Declaration of Helsinki, and the protocol was approved by the Social and Behavioral Sciences Institutional Review Board of the State University of New York at Buffalo (STUDY00002214).

### 2.2. Experimental Design and Procedures

Before participation, an overview of the study was provided. Eligible participants were randomized to one of four conditions in a 2 × 2 factorial design, with “EFT/ERT” and “matched/unmatched cue presentation” as the between-group variables. ERT controls for engaging in episodic memory and has the participant generate recent cues rather than future cues. Eligible participants completed one 45-min survey that included consent, cue creation depending on group assignment, a delay-discounting task, a demographics questionnaire, and future orientation measures. No deception was used in this research protocol.

In terms of episodic cue generation, depending on group assignment, participants generated either EFT or ERT cues. Within the EFT condition, participants were either assigned to an EFT-matched group, where the created cues later matched the time delays of the DD task, or to one of four EFT-unmatched groups, where the created cues were presented out of order to the time delays of the DD task (see Table 1 for cue presentation order). Regardless of EFT group assignment, there were no differences during the cue creation portion of the experiment. EFT groups were asked to list and describe future positive events that were going to take place, or could reasonably take place in 1 day, 1 week, 1 month, 6 months, and 2 years. Because positive events are more vivid than negative events, only positive events were used [23]. Participants in the EFT groups listed one future event that they were looking forward to per time period. The future events were matched to the five time delays of the DD task. Participants rated the salience (importance), valence (enjoyment), arousal (exciting), and vividness of each event on scales from “1—not at all” to “5—very much”. For events that were rated low in vividness (less than 3), the survey asked the participant if a different, more vivid event was available within the same time frame. Vividness of cues is important because previous researchers have found that the vividness of EFT cues predicts reduced DD [23].

The ERT groups were asked to list positive and enjoyable events that took place within the last 60 h (e.g., 12 h ago, 24 h ago, 36 h ago). Participants in the ERT-matched group created cues that were later matched to the time delays of the discounting task, while participants in the ERT-unmatched groups created cues that were later presented out of order to the way they would normally be paired with time delays of the DD task (i.e., sequentially starting with 12 h ago; see Table 1 for cue presentation order). All five groups first listed all past recent events for each specified time period (e.g., “12 h ago I enjoyed a cup of coffee at home”, “24 h ago I met my friend for lunch”). Salience, valence, arousal, and vividness (“1—not at all” to “5—very much”) ratings were collected for each event. Events that were rated low in vividness (less than 3) were queried to see if another, more vivid event for the same time period could be listed instead.

After listing events, both EFT and ERT groups were asked to provide further episodic details about each event. Specifically, participants were asked to imagine and describe who they were with, what they were doing, where they were, and how they were feeling at each event. These elaborations were incorporated into the original one-sentence event description to create five individualized cues that were roughly 3–6 sentences long. Before each of the five time delays of the DD task, the corresponding cue, depending on group assignment, was presented on the screen for participants to read out loud. After completing each of the trials of DD, participants were asked to rate each event on how frequently they thought of their event (“1—never” to “5—always”) and how vivid their event was (“1—not vivid at all” to “5— highly vivid”). An overall imagery score was calculated by averaging vividness and frequency scores into one variable that assessed the degree to which the participants thought of their episodic events during DD.

In terms of the delay discounting task, participants made choices between immediate and delayed hypothetical monetary rewards across five time delays. In each of the five delays, the immediate amounts were available now while delayed rewards were available 1 day, 1 week, 1 month, 6 months, and 2 years into the future. The value of the delayed reward remained fixed at $100 while the value of the immediate reward was adjusted in specific dollar increments based on response, until an indifference point was reached. The indifference point represents the equivalence point between choosing the immediate reward and the future reward. Participants were prompted to vividly imagine their EFT or ERT cue during the DD task by reading their event out loud before each of the five time delays. The cue was presented in large font on the computer screen prior to each time delay for participants to read out loud and was also embedded within the DD task so participants could keep the event in mind as they made their monetary choices. A different cue was presented for each time delay: The EFT-matched group imagined future events that corresponded to the time delay of the DD task, while the EFT-unmatched groups imagined a different future event during each time delay (Table 1). The ERT-groups imagined a different recent event during each time delay (Table 1). Discounting of rewards in the DD task was determined by calculating area under the curve (AUC) values. AUC is a method of measuring DD by calculating the area under the empirical discounting function [24]. Individual AUC scores can range from 0 (highest possible discounting) to 1 (no discounting). An attention check was embedded in the middle of the delay discounting task to ensure participants were reading instructions and making choices carefully. The question asked whether they would prefer $0.00 now or $100 in 6 months. If participants chose $0.00 now, it was considered a failure to pay attention to the task, and they would be removed from data analyses. No participants failed the attention check.

In terms of demographics, participant demographic information was collected (e.g. sex, age, race/ethnicity, family income, and educational level).

In terms of time orientation measures, the Future Orientation Scale (FOS) assesses the extent to which individuals consider the potential future outcomes of their current behavior and the extent to which they prefer long-term, as opposed to short-term goals [25]. The FOS was scored for three five-item subscales: time perspective (e.g., “Some people spend very little time thinking about how things might be in the future BUT Other people spend a lot of time thinking about how things might be in the future”); anticipation of future consequences (e.g., “Some people usually think about the consequences before they do something BUT Other people just act—they don’t waste time thinking about the consequences”); and planning ahead (e.g., “Some people like to plan things out one step at a time BUT Other people like to jump right into things without planning them out beforehand”). The respondent was asked to choose between the two descriptors and then rate whether it was “sort of true for me” or “really true for me”. Higher scores indicated higher future orientation. To assess for the time period most relevant to an individual’s finances, participants were asked the following question, “In planning your, or your family’s, saving and spending, which of the time periods is more important to you and your partner, if you have one?” The answer choices provided were categorical and ranged from not planning, planning for the next 6 months or less, the next year, the next 5 years, and the next 10 years or more. Financial planning was converted to a continuous variable for analysis using midpoints of the categories [26]. Subjective probability of living to age 75 was measured by asking, “What do you think are the chances you will live to be 75 or more? (where 0 means there is no chance you will live to 75 or more, and 100 means you will definitely live to 75 or more)” [26]. Higher values were indicative of greater future orientation. These measures of time orientation allowed for a comparison between groups and whether these measures moderated the effect of EFT on reducing DD.

### 2.3. Analytical Plan

Variables were examined for skew and kurtosis. Any variables with non-normal distributions were log-transformed. One-way analysis of variance (ANOVA) was used to check for baseline differences in continuous variables, and chi-square was used to check for differences in dichotomous variables. A 2 × 2 factorial ANOVA was used to compare between-group differences in DD. We examined the effects of orientation (EFT vs ERT) and type of episodic thinking (matched vs unmatched), and the interaction between these two factors. Partial eta-squared (η_p_^2^) effect sizes [27] were included. Variables that predicted outcome or differed between groups were entered as covariates. Measures of time orientation (FOS, subjective probability of living to the age of 75, and financial planning orientation) and ratings of arousal, valence, salience, and vividness were investigated as moderators of the type of episodic thinking or temporal perspective on DD. Post hoc comparisons were used to study differences in DD for participants randomized to EFT or ERT who engaged in matched versus unmatched episodic thoughts. Data analyses were completed using SYSTAT (Systat Software Inc., San Jose, CA, USA) [28].

## 3. Results

**Figure 1:**
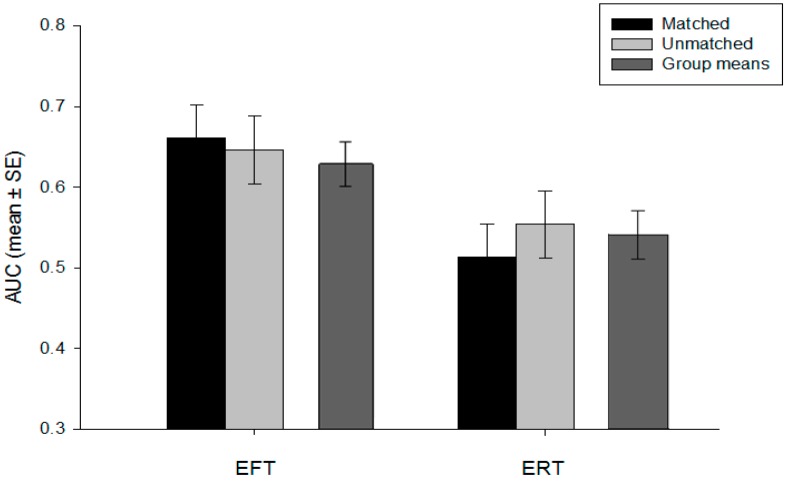
Area under the curve (AUC) values (mean ± SE) for the discounting of delayed rewards as a function of group.

No variables required data transformations due to non-normality. As shown in Table 2, household income was different across group, with post hoc contrasts showing the EFT-unmatched group having significantly higher incomes than both ERT groups (*p* < 0.05). No other significant group differences in demographic characteristics or time perspective were observed (i.e., sex, race; see Table 2). Analyses of the manipulation check data revealed no group differences in how much episodic events were thought about during the DD task (*F*(3, 156) = 0.27, *p* = 0.85) or how vivid the events were (*F*(3, 156) = 0.31, *p* = 0.82). Of the three time orientation measures, the only variable that was related to outcome was the financial planning time period, which was associated with DD (*r*(158) = 0.19, *p* = 0.01). With household income and financial planning included as covariates, there was a main effect of orientation (*F*(1, 154) = 8.21, *p* < 0.01; η_p_^2^ = 0.05), such that EFT groups showed less DD, or higher AUC values (M = 0.65, SD = 0.25), than the ERT groups (M = 0.53, SD = 0.27; Figure 1). No main effect of type of cue (matched vs. unmatched) was observed (*F*(1, 154) = 0.09, *p* = 0.76; η_p_^2^ < 0.001), and no interaction between orientation and type of cue was detected (*F*(1, 154) = 0.45, *p* = 0.50; η_p_^2^ = 0.003). Scores on the FOS, financial planning, and ratings of arousal, valence, salience, and vividness did not moderate the effect of time orientation on DD. There were no differences in discounting within the five variations of EFT cues (*F*(4, 75) = 0.27, *p* = 0.90; η_p_^2^ = 0.01), nor within the five variations of ERT cues (*F*(4, 75) = 0.51, *p* = 0.73; η_p_^2^ = 0.03).

## 4. Discussion

These results suggest that, regardless of whether EFT cues matched the time delays of the discounting task, EFT decreased DD. This is an important finding, as all previous research has used EFT cues that have matched the time delays [8,10,11], or have attempted to match the delays as much as possible [14,15,16]. This study shows that the EFT effect may not be dependent on matching the cues to the delay intervals in the DD task. The implication of this finding is that either the process of generating multiple EFT cues shifts present bias, or a future event within the time parameters of the DD task has the potential to shift discounting rates.

Previous research has compared one versus three cues, with the one cue being the cue that corresponded to the longest time delay that was studied in the task [16]. This study showed that matching the cues with the time delays was more effective in reducing DD than one cue matched to the longest delay. The current study compared the same number of cues, but varied whether the cues matched or did not match the time delays. Additionally, we found no differences between matched and unmatched EFT groups, suggesting cue presentation order was less important than the creation of multiple EFT cues. This has major implications for future EFT interventions, as the creation of multiple EFT cues not tied to specific time frames may be sufficient to change relevant behaviors. For example, participants may use general prospection to change overeating behaviors instead of creating EFT cues for specific time periods.

Both of the EFT groups created cues that were within the constraints of the DD task. It is possible that changes in temporal discounting may be different if the cues are not within the temporal window of the task. For example, a task might start comparing discounting rates at 1 month, while the EFT cues might be one, two, and four weeks ahead. Similarly, the task might only go up to one year, but the EFT cues are to project into the future for 2, 5, and 10 years. It would be interesting to know if EFT cues either prior to or after a given temporal window have the same effect as cues that are within the temporal window of the DD task.

Future studies may investigate whether simply the process of generating EFT cues alters discounting rates, or whether it is important to have episodic cues presented during the decision-making task. The mechanism by which EFT is thought to reduce DD is by increasing the value [12] or cognitive salience [13] of the delayed reward. Our results suggested that even when EFT cues were unmatched to the DD task, future needs were still deemed more valuable and important than immediate needs. This may suggest that the EFT cue generation procedure itself prompts individuals to view the future as more important and valuable, and this future activation prior to the DD task is enough to reduce discounting behavior. Engaging in EFT may lead to future-oriented details being incorporated into implicit memory, or the unconscious aspect of memory, such that even without cues presented during the task, delay-discounting rates are altered. In this way, EFT acts as a primer to making more future-oriented decisions. 

If the process of creating positive and vivid EFT cues may be enough to shift discounting behavior, a potential question becomes whether the repeated practice of generating EFT cues would enhance or reduce the EFT effect on DD. For example, would daily EFT cue generation over the course of multiple weeks result in an amplified EFT effect, or is a single session of EFT cue generation sufficient? This would be a valuable finding, as EFT could be incorporated into daily practice in order to influence distal decision-making in the absence of future cues. The results of this study may be important for future EFT interventions to reduce delay discounting and the maladaptive behaviors most associated with an inability to delay gratification. Imagining future events can promote decision-making that benefits one’s future self, and has important implications for many unhelpful behaviors. For the first time, this study has shown that the multiple EFT cues do not need to match the time delays of the DD task in order to be effective, suggesting the integral ingredient in EFT pertains to the cue creation, or presence of a future cue.

## Figures and Tables

**Table 1:** Cue presentation for episodic future thinking (EFT) and episodic recent thinking (ERT) groups depending on randomization.

**Delay Discounting** **Time Delay**	**EFT-Matched**	**EFT-Unmatched Group 1**	**EFT-Unmatched Group 2**	**EFT-Unmatched Group 3**	**EFT-Unmatched Group 4**
1 day	1 day	2 years	6 months	1 month	1 week
1 week	1 week	1 day	2 years	6 months	1 month
1 month	1 month	1 week	1 day	2 years	6 months
6 months	6 months	1 month	1 week	1 day	2 years
2 years	2 years	6 months	1 month	1 week	1 day
**Delay Discounting** **Time Delay**	**ERT-Matched**	**ERT-Unmatched Group 1**	**ERT-Unmatched Group 2**	**ERT-Unmatched Group 3**	**ERT-Unmatched Group 4**
1 day	12 h ago	60 h ago	48 h ago	36 h ago	24 h ago
1 week	24 h ago	12 h ago	60 h ago	48 h ago	36 h ago
1 month	36 h ago	24 h ago	12 h ago	60 h ago	48 h ago
6 months	48 h ago	36 h ago	24 h ago	12 h ago	60 h ago
2 years	60 h ago	48 h ago	36 h ago	24 h ago	12 h ago

**Table 2:** Participant characteristics.

	*N* (%)				
	EFT Matched (*n* = 40)	EFT Unmatched (*n* = 40)	ERT Matched (*n* = 40)	ERT Unmatched (*n* = 40)	*p*-Value
Sex					0.62
Male	19 (47.50)	24 (60.00)	19 (47.50)	22 (55.00)	
Female	21 (52.50)	16 (40.00)	21 (52.50)	18 (45.00)	
Race					0.74
Non-Minority	28 (70.00)	25 (62.50)	29 (72.50)	29 (72.50)	
Minority	12 (30.00)	15 (37.50)	11 (27.50)	11 (27.50)	
	**Mean (SD)**				
Education (years)	15.43 (1.75)	16.30 (1.62)	15.95 (1.85)	16.05 (1.85)	0.16
Age (years)	35.58 (9.68)	36.53 (9.28)	35.08 (7.46)	33.83 (11.05)	0.64
Household Income ($)	45,749.83 (41,564.90)	61,315.71 (42,691.39)	43,999.95 (23,944.45)	40,256.23 (23,562.83)	0.04 *
Future Orientation Scale					
Time Perspective	2.64 (0.34)	2.55 (0.28)	2.58 (0.32)	2.50 (0.48)	0.40
Anticipation of FC ^1^	2.33 (0.37)	2.33 (0.47)	2.44 (0.40)	2.22 (0.39)	0.13
Planning Ahead	2.50 (0.29)	2.43 (0.32)	2.51 (0.32)	2.35 (0.33)	0.10
Financial Planning (years)	2.63 (3.45)	2.73 (3.47)	3.50 (3.75)	3.36 (3.62)	0.61
Longevity (%)	75.95 (22.68)	73.05 (28.74)	73.25 (23.95)	78.55 (18.06)	0.70

^1^ Note: FC = future consequences, * *p* < 0.05.
